# Supplementary material for: Association between subjective tinnitus and cognitive performance: protocol for systematic review and meta-analysis
Source: BMJ Open. 2018 Aug 13;8(8):e023700. doi: 10.1136/bmjopen-2018-023700 (PMC6091911; doi:10.1136/bmjopen-2018-023700)
Supplement: Author's manuscript [file bmjopen-2018-023700.draft_revisions.pdf]

BMJ Open

BMJ Open is committed to open peer review. As part of this commitment we make the peer review history of every article we publish publicly available.

When an article is published we post the peer reviewers' comments and the authors' responses online. We also post the versions of the paper that were used during peer review. These are the versions that the peer review comments apply to.

The versions of the paper that follow are the versions that were submitted during the peer review process. They are not the versions of record or the final published versions. They should not be cited or distributed as the published version of this manuscript.

BMJ Open is an open access journal and the full, final, typeset and author-corrected version of record of the manuscript is available on our site with no access controls, subscription charges or pay-per-view fees (http://bmjopen.bmj.com).

If you have any questions on BMJ Open's open peer review process please email info.bmjopen@bmj.com

BMJ Open

The association between subjective tinnitus and cognitive performance: protocol for systematic review and meta-analysis

Journal:	*BMJ Open*
Manuscript ID	bmjopen-2018-023700
Article Type:	Protocol
Date Submitted by the Author:	19-Apr-2018

Complete List of Authors:	Clarke, Nathan; National Institute of Health Research Nottingham Biomedical Centre, Otology and Hearing Group; MRC Institute of Hearing Research<br>Akeroyd, Michael; MRC Institute of Hearing Research; University of Nottingham School of Medicine<br>Henshaw, Helen; University of Nottingham, School of Medicine; NIHR Nottingham Hearing Biomedical Research Unit, NIHR Nottingham Hearing Biomedical Research Unit<br>Hoare, Derek; University of Nottingham, NIHR Nottingham Hearing Biomedical Research Unit

Keywords:	Tinnitus, Cognition, Memory, Audiology < OTOLARYNGOLOGY, Attention, ENT

SCHOLARONE™
Manuscripts

The association between subjective tinnitus and cognitive performance: protocol for systematic review and meta-analysis

Nathan A Clarke [1, 2], Michael Akeroyd [1], Helen Henshaw [2] & Derek J Hoare [2]

[1.] *Medical Research Council Institute of Hearing Research, School of Medicine, The University of Nottingham, University Park, Nottingham, UK*

[2.] *National Institute of Health Research Nottingham Biomedical Research Centre, Otology and Hearing Group, Division of Clinical Neuroscience, University of Nottingham, Nottingham, United Kingdom*

Correspondence: Nathan Clarke. National Institute of Health Research Nottingham Biomedical Research Centre, Otology and Hearing Group, Division of Clinical Neuroscience, University of Nottingham, Nottingham, United Kingdom. E-mail: nathan.clarke@nottingham.ac.uk

Nathan Andrew Clarke https://orcid.org/0000-0002-8636-1872
Michael Akeroyd https://orcid.org/0000-0002-7182-9209
Helen Henshaw https://orcid.org/0000-0002-0547-4403
Derek James Hoare https://orcid.org/0000-0002-8768-1392

WORD COUNT: 1050 (excluding title page, abstract, references, figures and tables)

For peer review only - http://bmjopen.bmj.com/site/about/guidelines.xhtml

ABSTRACT

Introduction:

Subjective tinnitus is very common and has a number of comorbid associations including depression, sleep disturbance and concentration difficulties. Concentration difficulties may be observable in people with tinnitus through poorer behavioural performance in tasks thought to measure specific cognitive domains such as attention and memory (i.e. cognitive performance). Several reviews have discussed the association between tinnitus and cognition, however, none to date have investigated the association between tinnitus and cognitive performance through meta-analysis. Furthermore, there has been little overlap between sets of studies that have been included in previous reviews, potentially contributing to the typically mixed findings that are reported.

Methods and analysis:

This systematic review aims to comprehensively review the literature and quantitatively synthesize relevant data to determine associations between subjective tinnitus and cognitive performance. Methods are reported according to Preferred Reporting Items for Systematic Reviews and Meta-Analyses Protocols (PRISMA-P). All study designs will be eligible for inclusion with no date restrictions on searches. Studies eligible for inclusion must contain adult participants (≥18 years) with self-reported, subjective tinnitus and a behavioural measure of cognitive performance. Meta-analysis will be reported via correlation for the association between tinnitus and cognitive performance.

Ethics and dissemination:

No ethical issues are foreseen. Findings will be reported in a student thesis, at national and international ENT/audiology conferences and by peer-reviewed publication.

Systematic review registration number:

PROSPERO 2018 CRD42018085528.

Strengths and limitations of this study

- This systematic review and meta-analysis protocol poses a clearly formulated research question and methodology to investigate a common clinical complaint of tinnitus patients; peer-reviewed evidence to date will be synthesised.
- Grey literature and dissertation abstracts will not be included

Introduction

Subjective tinnitus is the experience of sound in the ears or head in the absence of an external source and is commonly considered a symptom of damage within the auditory system.[1] Most individuals who experience tinnitus do not find it bothersome but a significant proportion are disturbed by it, often reporting a variety of adverse comorbid associations including anxiety, depression, disturbed sleep, or concentration difficulties. [2-4] Concentration difficulties can be conceptualised as failures of

cognitive performance in various domains such as attention and memory [5, 6] and previous research has implicated tinnitus as impacting cognitive domains including executive functions, attention and working memory. [7-9] Investigating the potential impact of tinnitus on cognitive performance is further complicated by the strong associations that tinnitus shares with depression and anxiety, which can independently negatively impact cognitive performance. [4, 10, 11]

Several reviews have explored the relationship between tinnitus and cognition.[12-15] We note that all of these are narrative reviews; no review has quantitatively synthesised the literature specifically concerning tinnitus and behaviourally measured cognitive performance. An overview of previous reviews and their methodologies is provided in Table 1.

Table 1. Overview of previous reviews investigating cognition and tinnitus

Study	Databases searched	Example search strategy provided	Number of records retrieved during database search	Number of studies included in review	Synthesis of association between cognition and tinnitus
Trevis et al. (2017)	PsycINFO, MedLine	(1) tinnitus AND psych* (all fields), (2) tinnitus AND mood (all fields), (3) tinnitus AND depress* (all fields), (4) tinnitus AND anx* OR stress (all fields)	725	35	Meta-analysis of 'psychological functioning' in tinnitus participants. Narrative review of association between 'cognitive functioning' (n = 16); i.e. behavioural cognitive task performance and chronic tinnitus. awareness and severity
Mohamad et al. (2016)	PubMed	((((tinnitus[Title]) AND cogniti*[Title])) OR ((tinnitus[Title]) AND attention[Title])) OR ((tinnitus[Title]) AND memory[Title])	65	9	Narrative review of 'behavioral research' addressing the impact of tinnitus and its severity of various aspects of cognitive performance in domains of working memory and attention.
Tegg-Quinn et al. (2016)	PubMed, MedLine, CINAHL, SCOPUS, EMBASE	(tinnitus) and (cognition OR memory OR attention OR concentration OR cognitive function OR mental activity) NOT (infant OR child OR adolescent OR paediatric OR animal OR balance OR hyperacusis OR implant OR pharmaceutical OR drugs)	2236	18	Narrative review of behavioural cognitive tasks, electrophysiological correlates of cognition and self-reported cognitive function measures.
Andersson and McKenna (2006)	Not reported	Not reported	Not reported	Not reported	Narrative review of 'cognitive deficits' (i.e. behavioural cognitive tasks), 'cognitive bias' and 'concious appraisal of tinnitus'.

Andersson and McKenna[12] were the first to review the relationship between cognition and tinnitus, detailing three separate but related lines of cognitive research. The strands of research included neuropsychological studies involving attention, cognitive bias (concerning selective attention and memory) and appraisal (i.e. conscious recollection) of tinnitus. Tegg-Quinn et al.[13] performed a systematic review of all studies pertaining to the impact of tinnitus upon cognition in adults. The review described studies that included both behavioural and electrophysiological measures and concluded that tinnitus impairs cognition through impacting on executive control of attention. Mohamad et al.[14] performed a narrative review of the behavioural evidence concerning the

For peer review only - http://bmjopen.bmj.com/site/about/guidelines.xhtml

consequences of tinnitus and its severity on cognition. They concluded that there was suggestive evidence for tinnitus being associated with poorer performance in behavioural tasks attempting to measure executive attention, selective attention and working memory. Trevis et al.[15] performed a systematic review and meta-analysis of psychological functioning in chronic tinnitus. The authors predominantly investigated the presence and severity of tinnitus in relation to emotional wellbeing through meta-analysis, while cognitive function (i.e. cognitive performance) was described through a narrative review. Of the recent reviews that discuss cognition through behavioural performance in tinnitus participants, there is a notable lack of overlap in the studies that met criteria for inclusion in the final reviews; e.g. Mohamad et al.[14] reviewed nine studies and Trevis et al.[15] reviewed a total of thirty-five studies (with sixteen of these concerning cognitive performance), however, only three studies were included in both reviews. Therefore, previous work has essentially investigated the association between tinnitus and cognitive performance with different datasets. Like any statistical technique, meta-analysis is only as robust as the data that is inputted. It is, therefore, essential to include as much relevant data as possible - through a comprehensive search strategy - to ensure that conclusions are based on all evidence for an effect of tinnitus on specific cognitive domains.

Tinnitus is a symptom of heterogeneous and often unknown aetiologies. It is, therefore, inherently difficult to define and specify within the context of a systematic review. Different inclusion criteria and working definitions of tinnitus are likely to significantly influence the records included within a review. For example, Trevis et al.[15] defined ‘chronic tinnitus’ as participants who had experienced tinnitus for at least one month. An alternative approach would be to not attempt to temporally specify a population, but rather investigate this variable through further quantitative analysis if feasible. With regards to domains of cognitive performance, the aforementioned reviews have implicated tinnitus as impacting executive attention, although the evidence is not conclusive: additional domains of cognitive performance are also potentially associated with tinnitus, including selective attention and working memory[12-15]. A promising approach to foster empirically valid insights into any association between cognition and subjective tinnitus is through evaluating and categorising tests of cognitive performance according to the theoretical constructs that they are thought to measure[16]. Any associations between categorised measures of cognitive performance and subjective tinnitus can then be subjected to meta-analysis in order to understand which cognitive domains are associated with tinnitus, as well as the best estimate of any such effect. To summarise, although several authors have reviewed associations between tinnitus and cognitive performance assessed through behavioural measures, these have been through narrative syntheses, discussing different sets of studies derived from different search strategies. A comprehensive, quantitative investigation of the association between tinnitus and cognitive performance, building on earlier efforts in this field and exploring the underlying theoretical domains of cognition involved, is therefore both necessary and timely.

For peer review only - http://bmjopen.bmj.com/site/about/guidelines.xhtml

The primary aim of this work is to comprehensively review the literature and synthesize relevant data to determine the associations between self-reported subjective tinnitus and cognitive task performance in adults. If possible, a secondary examination of patient characteristics (e.g. age or gender) or commonly used patient-reported outcomes (e.g. depression or anxiety) and their influence on any association between subjective tinnitus and cognitive performance will also be conducted.

Methods and Analysis

The methodology of this review is reported in accordance with the Preferred Reporting Items for Systematic Reviews and Meta-Analysis Protocols (PRISMA-P) checklist[17]. Specified roles of named authors are identified throughout the review protocol.

Eligibility criteria

Only published or in-press, peer-reviewed journal articles will be considered. Articles that are not written in English or have no English language translation available will be excluded as the review team does not have resource available to support translation. No date restriction will be applied.

Review inclusion criteria are specified according to Participant, Intervention (or Interest), Comparator, Outcome and Setting (PICOS) characteristics.

Participants: Studies including adults (≥18 years) with subjective tinnitus. Studies that include both children (<18 years) and adults will be excluded, unless the adult data are reported separately.

Intervention/Interest: Subjective tinnitus (via self-report, tinnitus severity scale, item or established tinnitus questionnaires).

Comparator: A minimum of one established measure of cognitive performance (behavioural or self-report).

Outcome measures: An association between tinnitus and cognitive performance. Where available, data for associations between subjective tinnitus and additional potential moderator variables will be extracted, such as measures of anxiety or depression.

Study design: Any type of study design and setting will be considered.

Information Sources and Search strategy

For peer review only - http://bmjopen.bmj.com/site/about/guidelines.xhtml

A systematic search strategy will be employed to identify completed, peer-reviewed journal articles from the following bibliographic databases: MEDLINE (via Ovid SP), EMBASE (via Ovid SP), PsycINFO (via Ovid SP), ASSIA (via ProQuest), CINAHL (via EBSCO Host), Scopus, PubMed, and Web of Science (Science and Social Science Citation Index). Searches will be performed during 01/02/18 – 01/03/18.

The search terms to be used in this systematic review were identified using free text, controlled vocabularies (i.e. Medical Subject Headings - MeSH and CINAHL Headings), literature review, expert opinion, and scrutiny of test search results. The following search strategy will be used for PubMed, which will then be adapted for other databases to be searched:

(“tinnitus”[MeSH] OR “tinnitus”[tiab] OR “phantom sound*”[tiab] OR “ringing”[tiab] OR “buzzing”[tiab]) AND (“cognition”[MeSH] OR “cogniti*”[tiab] OR “memory”[tiab] or “attention*”[tiab] OR “executive”[tiab])

Data management

NAC will be responsible for data management. Covidence online systematic review software (https://www.covidence.org) will be used for article screening and data management throughout the review. All articles identified through the search process will be recorded digitally and will be trackable through the data screening and extraction processes. Articles excluded at the full text screening stage will have justification for exclusion noted. Included articles will be assigned a unique study identification code, enabling it to be linked to its corresponding full text and data collection sheet.

Article selection process

NAC, DH, and MA will independently screen titles and abstracts of records retrieved from searches such that all records are independently screened by two reviewers. Records that meet the specified inclusion criteria will then be taken forward to full text screening, as well as records where there is too little information available to make a decision to exclude. All full texts selected will be independently screened by two reviewers who will resolve any discrepancies in which records are included. Where discrepancies for inclusion are not resolved by the two reviewers, a third reviewer will adjudicate. If necessary, study authors will be contacted to request additional information that may help ascertain suitability for inclusion.

Data extraction process

Data extraction will be performed using a customised form via Covidence. Relevant guidance notes will be created and disseminated to review team members by NAC prior to commencement. The data form and guidance notes will be piloted by NAC and HH. All included records will be subject to data

For peer review only - http://bmjopen.bmj.com/site/about/guidelines.xhtml

extraction. Data from each included record will be extracted independently by two reviewers and the results compared. Any disagreements arising will be resolved through discussion or the involvement of a third reviewer.

Data items

The data collection form will include various fields corresponding to study type, population, reported effect size, and any other relevant study findings. Study authors will be contacted if there are relevant missing data. Data will be approximated from figures for instances where it can only be estimated, using software such as WebPlotDigitizer (http://arohatgi.info/WebPlotDigitizer/app/). Disagreements regarding numerical data extracted from figures will discussed by investigators and/or resolved by averaging.

Risk of bias in individual studies

NAC, HH, and DH (two reviewers per record) will independently assess the risk of bias using relevant items adapted from the Critical Appraisal Skills Programme (CASP) Case Control Checklist (https://casp-uk.net/casp-tools-checklists/).

Data synthesis

The effect of interest is the association between subjective tinnitus and measures of cognitive performance expressed as correlation. Where possible, results not expressed as correlations (e.g. mean differences between groups) will be calculated as appropriate correlations, such as point-biserial or polyserial correlation coefficients.[18] Bias corrections will be applied to all derived correlation coefficients prior to pooling.[19] Where possible, missing effect sizes will be calculated from reported test statistics such as standard deviations or t-values[18]. Narrative synthesis will be undertaken for records where appropriate effect sizes cannot be obtained.

Assessment of heterogeneity

The ratio of observed variation to within-study variance will be assessed with the Q-statistic, used to test the null hypothesis of homogeneity of effect sizes. The I^2 statistic will provide a further index of heterogeneity across studies. If the apparent heterogeneity across studies exceeds 50%, potential causes of heterogeneity will be explored through subgroup analysis. The Tau-squared statistic will also be used to assess the amount of heterogeneity where a random-effects model is fitted to the data. A Baujat plot will be used as a graphical means of identifying studies that contribute excessively to any observed heterogeneity and also provide insight into potential moderating variables that contribute to heterogeneity across studies[20].

For peer review only - http://bmjopen.bmj.com/site/about/guidelines.xhtml

Subgroup analyses

Potential contributors to heterogeneity across studies will be explored through subgroup analysis (i.e. sensitivity analyses, moderator analysis or meta-regression). Variables of potential interest for subgroup analyses include participant age, gender, presence of hearing impairment and presence of anxiety or depression, or study design aspects, such as inclusion of a control group.

Assessment of reporting bias

The influence of reporting bias through potentially unpublished results (i.e. publication bias) will be explored via funnel plots, rank correlation test and/or Egger's regression test.

Ethics and dissemination

No ethical issues are foreseen in this systematic review. Reports will be guided by the Preferred Reporting Items for Systematic Reviews and Meta-Analysis guidelines[21]. Various dissemination strategies will be employed, that will likely include: journal article and PhD thesis (NC) made available via an institutional repository, results being reported at national and international academic conferences, and public and patient engagement (e.g. articles written for relevant non-specialist audiences).

Summary

A protocol is described for a -systematic review and meta-analysis to determine a best estimate of the association between subjective tinnitus and cognitive performance in adults. To date, no review has comprehensively explored the veracity of an association through application of quantitative analyses of all available peer-reviewed data. The outlined approach will facilitate an understanding of the potential impact of tinnitus upon cognition, underpinned by relevant cognitive theory. An increased understanding of the relationship between subjective tinnitus and cognitive performance will eventually improve tinnitus sub-typing and inform therapeutic methods[22-25] for example, it may be possible to deliver cognitive training paradigms in a targeted manner.

Acknowledgements

Thanks to Oliver Zobay for statistical advice.

For peer review only - http://bmjopen.bmj.com/site/about/guidelines.xhtml

NAC is supported by a Medical Research Council studentship.
MA is supported by the Medical Research Council (grant number MC_UU_00010/3).
HH and DJH are funded by the National Institute for Health Research (NIHR) Biomedical Research Centre programme. The views expressed are those of the authors and not necessarily those of the NIHR, the NHS, or the Department of Health and Social Care.

References

1. Sedley W, Friston KJ, Gander PE, *et al.* An Integrative Tinnitus Model Based on Sensory Precision. *Trends Neurosci* 2016;**39**:799–812.
2. Tyler RS, Baker LJ. Difficulties experienced by tinnitus sufferers. *J Speech Hear Disord* 1983;**48**:150–4.
3. Andersson G, Lyttkens L, Larsen HC. Distinguishing levels of tinnitus distress. *Clin Otolaryngol Allied Sci* 1999;**24**:404–10.
4. Bhatt JM, Bhattacharyya N, Lin HW. Relationships between tinnitus and the prevalence of anxiety and depression. *Laryngoscope* 2017;**127**:466–9.
5. Petersen SE, Posner MI. The attention system of the human brain: 20 years after. *Annu Rev Neurosci* 2012;**35**:73–89.
6. Baddeley A. Working memory. *Curr Biol* 2010;**20**:R136–40.
7. Hallam RS, McKenna L, Shurlock L. Tinnitus impairs cognitive efficiency. *Int J Audiol* 2004;**43**:218–26.
8. Rossiter S, Stevens C, Walker G. Tinnitus and its effect on working memory and attention. *J Speech Lang Hear Res* 2006;**49**:150–60.
9. Heeren A, Maurage P, Perrot H, *et al.* Tinnitus specifically alters the top-down executive control sub-component of attention: evidence from the Attention Network Task. *Behav Brain Res* 2014;**269**:147–54.
10. Robinson OJ, Vytal K, Cornwell BR, *et al.* The impact of anxiety upon cognition: perspectives from human threat of shock studies. *Front Hum Neurosci* 2013;**7**:203.
11. Lee RSC, Hermens DF, Porter MA, *et al.* A meta-analysis of cognitive deficits in first-episode Major Depressive Disorder. *J Affect Disord* 2012;**140**:113–24.

For peer review only

For peer review only - http://bmjopen.bmj.com/site/about/guidelines.xhtml

12. Andersson G, McKenna L. The role of cognition in tinnitus. *Acta Otolaryngol Suppl* 2006;:39–43.

13. Mohamad N, Hoare DJ, Hall DA. The consequences of tinnitus and tinnitus severity on cognition: A review of the behavioural evidence. *Hear Res* 2016;**332**:199–209.

14. Tegg-Quinn S, Bennett RJ, Eikelboom RH, *et al.* The impact of tinnitus upon cognition in adults: A systematic review. *Int J Audiol* 2016;**55**:533–40.

15. Trevis KJ, McLachlan NM, Wilson SJ. A systematic review and meta-analysis of psychological functioning in chronic tinnitus. *Clin Psychol Rev* Published Online First: 30 December 2017. doi:10.1016/j.cpr.2017.12.006

16. Dryden A, Allen HA, Henshaw H, *et al.* The Association Between Cognitive Performance and Speech-in-Noise Perception for Adult Listeners: A Systematic Literature Review and Meta-Analysis. *Trends in Hearing* Published Online First: 14 December 2017. doi:10.1177/2331216517744675

17. Shamseer L, Moher D, Clarke M, *et al.* Preferred reporting items for systematic review and meta-analysis protocols (PRISMA-P) 2015: elaboration and explanation. *BMJ* 2015;**349**:g7647.

18. Lipsey MW, Wilson DB. *Practical Meta-Analysis*. SAGE Publications 2000.

19. Hunter JE, Schmidt FL. *Methods of Meta-Analysis: Correcting Error and Bias in Research Findings*. SAGE 2004.

20. Baujat B, Mahé C, Pignon J-P, *et al.* A graphical method for exploring heterogeneity in meta-analyses: application to a meta-analysis of 65 trials. *Stat Med* 2002;**21**:2641–52.

21. Moher D, Liberati A, Tetzlaff J, *et al.* Preferred reporting items for systematic reviews and meta-analyses: the PRISMA statement. *PLoS Med* 2009;**6**:e1000097.

22. Hoare DJ, Stacey PC, Hall DA. The efficacy of auditory perceptual training for tinnitus: a systematic review. *Ann Behav Med* 2010;**40**:313–24.

23. Baune BT, Renger L. Pharmacological and non-pharmacological interventions to improve cognitive dysfunction and functional ability in clinical depression--a systematic review. *Psychiatry Res* 2014;**219**:25–50.

24. Ferguson M, Henshaw H. How Does Auditory Training Work? Joined-Up Thinking and Listening. *Semin Hear* 2015;**36**:237–49.

For peer review only - http://bmjopen.bmj.com/site/about/guidelines.xhtml

324 25. Barrett DJK, Pilling M. Evaluating the Precision of Auditory Sensory Memory as an Index
325 Intrusion in Tinnitus. *Ear Hear* 2017;**38**:262–5.

326 NAC is the guarantor of the review (CRD42018085528). NAC led on the development of all sections
327 of the review protocol. DH, HH & MA read, developed and provided feedback for all sections of the
328 review protocol and approved the final manuscript.

329

For peer review only

For peer review only - http://bmjopen.bmj.com/site/about/guidelines.xhtml

PRISMA-P 2015 Checklist

This checklist has been adapted for use with protocol submissions to *Systematic Reviews* from Table 3 in Moher D et al: Preferred reporting items for systematic review and meta-analysis protocols (PRISMA-P) 2015 statement. *Systematic Reviews* 2015 **4**:1

Section/topic	#	Checklist item	Information reported		Line number(s)
			Yes	No	
**ADMINISTRATIVE INFORMATION**					
**Title**					
Identification	1a	Identify the report as a protocol of a systematic review	☒	☐	1
Update	1b	If the protocol is for an update of a previous systematic review, identify as such	☐	☐	N/A
**Registration**	2	If registered, provide the name of the registry (e.g., PROSPERO) and registration number in the Abstract	☒	☐	53
**Authors**					
Contact	3a	Provide name, institutional affiliation, and e-mail address of all protocol authors; provide physical mailing address of corresponding author	☒	☐	6-15
Contributions	3b	Describe contributions of protocol authors and identify the guarantor of the review	☒	☐	325
**Amendments**	4	If the protocol represents an amendment of a previously completed or published protocol, identify as such and list changes; otherwise, state plan for documenting important protocol amendments	☐	☐	N/A
**Support**					
Sources	5a	Indicate sources of financial or other support for the review	☒	☐	266-270
Sponsor	5b	Provide name for the review funder and/or sponsor	☒	☐	266-270
Role of sponsor/funder	5c	Describe roles of funder(s), sponsor(s), and/or institution(s), if any, in developing the protocol	☒	☐	266-270
**INTRODUCTION**					
**Rationale**	6	Describe the rationale for the review in the context of what is already known	☒	☐	33-40, 62-127
**Objectives**	7	Provide an explicit statement of the question(s) the review will address with reference to	☒	☐	128-132, 147-159

BioMed Central
The Open Access Publisher

For peer review only

Section/topic	#	Checklist item	Information reported		Line number(s)
			Yes	No	
		participants, interventions, comparators, and outcomes (PICO)			
**METHODS**					
**Eligibility criteria**	8	Specify the study characteristics (e.g., PICO, study design, setting, time frame) and report characteristics (e.g., years considered, language, publication status) to be used as criteria for eligibility for the review	☒	☐	141-159
**Information sources**	9	Describe all intended information sources (e.g., electronic databases, contact with study authors, trial registers, or other grey literature sources) with planned dates of coverage	☒	☐	162-165
**Search strategy**	10	Present draft of search strategy to be used for at least one electronic database, including planned limits, such that it could be repeated	☒	☐	170-172
STUDY RECORDS					
Data management	11a	Describe the mechanism(s) that will be used to manage records and data throughout the review	☒	☐	175-181
Selection process	11b	State the process that will be used for selecting studies (e.g., two independent reviewers) through each phase of the review (i.e., screening, eligibility, and inclusion in meta-analysis)	☒	☐	184-191
Data collection process	11c	Describe planned method of extracting data from reports (e.g., piloting forms, done independently, in duplicate), any processes for obtaining and confirming data from investigators	☒	☐	194-199
**Data items**	12	List and define all variables for which data will be sought (e.g., PICO items, funding sources), any pre-planned data assumptions and simplifications	☒	☐	202-207, 235-237
**Outcomes and prioritization**	13	List and define all outcomes for which data will be sought, including prioritization of main and additional outcomes, with rationale	☒	☐	215-221, 234-237
**Risk of bias in individual studies**	14	Describe anticipated methods for assessing risk of bias of individual studies, including whether this will be done at the outcome or study level, or both; state how this information will be used in data synthesis	☒	☐	209-212, 239-241
DATA					
**Synthesis**	15a	Describe criteria under which study data will be quantitatively synthesized	☒	☐	226
	15b	If data are appropriate for quantitative synthesis, describe planned summary measures, methods of handling data, and methods of combining data from studies, including any planned exploration of consistency (e.g., I^2, Kendall's tau)	☒	☐	215-221, 224-229
	15c	Describe any proposed additional analyses (e.g., sensitivity or subgroup analyses, meta-	☒	☐	233-237

BioMed Central
The Open Access Publisher

For peer review only

3

Section/topic	#	Checklist item	Information reported Yes	Information reported No	Line number(s)
		regression)			
	15d	If quantitative synthesis is not appropriate, describe the type of summary planned	☒	☐	221-222
**Meta-bias(es)**	16	Specify any planned assessment of meta-bias(es) (e.g., publication bias across studies, selective reporting within studies)	☒	☐	240-242
**Confidence in cumulative evidence**	17	Describe how the strength of the body of evidence will be assessed (e.g., GRADE)	☒	☐	210-212

For peer review only

BioMed Central The Open Access Publisher

BMJ Open

The association between subjective tinnitus and cognitive performance: protocol for systematic review and meta-analysis

Journal:	*BMJ Open*
Manuscript ID	bmjopen-2018-023700.R1
Article Type:	Protocol
Date Submitted by the Author:	03-Jul-2018
Complete List of Authors:	Clarke, Nathan; National Institute of Health Research Nottingham Biomedical Centre, Otology and Hearing Group; MRC Institute of Hearing Research<br>Akeroyd, Michael; MRC Institute of Hearing Research; University of Nottingham School of Medicine<br>Henshaw, Helen; University of Nottingham, School of Medicine; NIHR Nottingham Hearing Biomedical Research Unit, NIHR Nottingham Hearing Biomedical Research Unit<br>Hoare, Derek; University of Nottingham, NIHR Nottingham Hearing Biomedical Research Unit
**Primary Subject Heading**:	Ear, nose and throat/otolaryngology
Secondary Subject Heading:	Ear, nose and throat/otolaryngology
Keywords:	Tinnitus, Cognition, Memory, Audiology < OTOLARYNGOLOGY, Attention, ENT

SCHOLARONE™
Manuscripts

The association between subjective tinnitus and cognitive performance: protocol for systematic review and meta-analysis

Nathan A. Clarke [1, 2, 3], Michael A. Akeroyd [1,3], Helen Henshaw [2] & Derek J. Hoare [2]

[1.] Medical Research Council Institute of Hearing Research, School of Medicine, The University of Nottingham, University Park, Nottingham, UK

[2.] National Institute of Health Research Nottingham Biomedical Research Centre,

[3]. Hearing Sciences, Division of Clinical Neurosciences, School of Medicine, The University of Nottingham, University Park, Nottingham UK

Correspondence: Nathan Clarke. National Institute of Health Research Nottingham Biomedical Research Centre, Otology and Hearing Group, Division of Clinical Neuroscience, University of Nottingham, Nottingham, United Kingdom. E-mail: nathan.clarke@nottingham.ac.uk

Nathan Andrew Clarke https://orcid.org/0000-0002-8636-1872
Michael Akeroyd https://orcid.org/0000-0002-7182-9209
Helen Henshaw https://orcid.org/0000-0002-0547-4403
Derek James Hoare https://orcid.org/0000-0002-8768-1392

WORD COUNT: 1373 (excluding title page, abstract, references, figures and tables)

For peer review only

For peer review only - http://bmjopen.bmj.com/site/about/guidelines.xhtml

ABSTRACT

Introduction:

Subjective tinnitus is very common and has a number of comorbid associations including depression, sleep disturbance and concentration difficulties. Concentration difficulties may be observable in people with tinnitus through poorer behavioural performance in tasks thought to measure specific cognitive domains such as attention and memory (i.e. cognitive performance). Several reviews have discussed the association between tinnitus and cognition, however, none to date have investigated the association between tinnitus and cognitive performance through meta-analysis with reference to an established theoretical taxonomy. Furthermore, there has been little overlap between sets of studies that have been included in previous reviews, potentially contributing to the typically mixed findings that are reported.

Methods and analysis:

This systematic review aims to comprehensively review the literature using an established theoretical taxonomy and quantitatively synthesize relevant data to determine associations between subjective tinnitus and cognitive performance. Methods are reported according to Preferred Reporting Items for Systematic Reviews and Meta-Analyses Protocols (PRISMA-P). All study designs will be eligible for inclusion with no date restrictions on searches. Studies eligible for inclusion must contain adult participants (≥18 years) with subjective tinnitus and a behavioural measure of cognitive performance. Meta-analysis will be reported via correlation for the association between tinnitus and cognitive performance.

Ethics and dissemination:

No ethical issues are foreseen. Findings will be reported in a student thesis, at national and international ENT/audiology conferences and by peer-reviewed publication.

Systematic review registration number:

PROSPERO 2018 CRD42018085528.

Funding statement:

NAC is supported by a Medical Research Council studentship.

MAA is supported by the Medical Research Council (grant number MC_UU_00010/3).

HH and DJH are funded by the National Institute for Health Research (NIHR) Biomedical Research Centre programme. The views expressed are those of the authors and not necessarily those of the NIHR, the NHS, or the Department of Health and Social Care.

Contributors: NAC led on the development of all sections of the review protocol. DJH, HH & MAA developed and provided feedback for all sections of the review protocol and approved the final manuscript.

Competing interests: None declared.

For peer review only - http://bmjopen.bmj.com/site/about/guidelines.xhtml

Strengths and limitations of this study

- This systematic review and meta-analysis protocol poses a clearly formulated research question and methodology to investigate a common clinical complaint of tinnitus patients; peer-reviewed evidence to date will be synthesised.
- This protocol details a comprehensive quantitative synthesis and inclusion of potential *a priori* moderator variables
- Synthesis will be clearly structured according to an established cognitive theoretical framework
- Grey literature and dissertation abstracts will not be included

Introduction

Tinnitus refers to the common experience of sound in the ears or head in the absence of an external source. It is commonly considered a symptom of damage within the auditory system [1]. Objective tinnitus involves sound with a known, non-central aetiology such as vascular abnormalities; it may be detected by an observer through auscultation. Objective tinnitus may be treated once the source of the aetiology has been identified and is therefore not of primary interest within this review [2]. Subjective tinnitus (hereafter discussed but simply referred to as 'tinnitus') involves sound of unknown aetiology. Most individuals who experience tinnitus do not find it bothersome but a significant proportion are disturbed by it, often reporting a variety of adverse comorbid associations including anxiety, depression, disturbed sleep, or concentration difficulties[3–5]. Concentration difficulties can be conceptualised as failures of cognitive performance expressed behaviourally (sometimes called objective cognition[6]) in various domains such as attention and memory[7,8]. Previous research has implicated tinnitus as negatively impacting cognitive performance in domains including executive functions, attention and working memory [9–11].Furthermore, a link between subjective perception of cognitive performance, or subjective cognitive complaints (SCC), has also been suggested [12]. Investigating the potential impact of tinnitus on cognitive performance is further complicated by the strong associations that tinnitus shares with depression and anxiety, which can independently negatively impact cognitive performance [5,13,14].

Several reviews have explored the relationship between tinnitus and cognition generally [12,15–17]. We note that all of these are narrative reviews; no review has quantitatively synthesised the literature specifically concerning tinnitus and behaviourally measured cognitive performance. An overview of previous reviews and their methodologies is provided in Table 1.

Table 1. Overview of previous reviews investigating cognition and tinnitus

For peer review only - http://bmjopen.bmj.com/site/about/guidelines.xhtml

Study	Databases searched	Example search strategy provided	Number of records retrieved during database search	Number of studies included in review	Synthesis of association between cognition and tinnitus
Trevis et al. (2018)	PsycINFO, MedLine	(1) tinnitus AND psych* (all fields), (2) tinnitus AND mood (all fields), (3) tinnitus AND depress* (all fields), (4) tinnitus AND anx* OR stress (all fields)	725	35	Meta-analysis of 'psychological functioning' in tinnitus participants. Narrative review of association between 'cognitive functioning' (n = 16); i.e. behavioural cognitive task performance and chronic tinnitus. awareness and severity
Mohamad et al. (2016)	PubMed	((((tinnitus[Title]) AND cogniti*[Title])) OR ((tinnitus[Title]) AND attention[Title])) OR ((tinnitus[Title]) AND memory[Title])	65	9	Narrative review of 'behavioural research' addressing the impact of tinnitus and its severity of various aspects of cognitive performance in domains of working memory and attention.
Tegg-Quinn et al. (2016)	PubMed, MedLine, CINAHL, SCOPUS, EMBASE	(tinnitus) and (cognition OR memory OR attention OR concentration OR cognitive function OR mental activity) NOT (infant OR child OR adolescent OR paediatric OR animal OR balance OR hyperacusis OR implant OR pharmaceutical OR drugs)	2236	18	Narrative review of behavioural cognitive tasks, electrophysiological correlates of cognition and self-reported cognitive function measures.
Andersson and McKenna (2006)	MedLine and Psychological Abstracts	Not reported	Not reported	Not reported	Narrative review of 'cognitive deficits' (i.e. behavioural cognitive tasks), 'cognitive bias and 'conscious appraisal of tinnitus'.

For peer review only - http://bmjopen.bmj.com/site/about/guidelines.xhtml

Andersson and McKenna [15] were the first to review the relationship between cognition and tinnitus, detailing three separate but related lines of cognitive research. The strands of research included neuropsychological studies involving attention, cognitive bias (concerning selective attention and memory) and appraisal (i.e. conscious recollection) of tinnitus. Tegg-Quinn et al. [16] performed a systematic review of all studies pertaining to the impact of tinnitus upon cognition in adults. The review described studies that included behavioural, electrophysiological and SCC measures. The authors concluded that tinnitus impairs cognition by adversely impacting the executive control of attention. Mohamad et al. [12] performed a narrative review of the behavioural evidence concerning the consequences of tinnitus and its severity on cognition. They concluded that there was suggestive evidence for tinnitus being associated with poorer performance in behavioural tasks attempting to measure executive attention, selective attention and working memory. This review also examined the proposed relationship between cognitive performance and SCC in individuals with tinnitus. They reported that their data was insufficient to form conclusions and recommended further investigation of the relationship. Trevis et al.[17] performed a systematic review and meta-analysis of psychological functioning in chronic tinnitus. The authors predominantly investigated the presence and severity of tinnitus in relation to emotional wellbeing through meta-analysis, while cognitive function (i.e. cognitive performance) was described through a narrative review. To summarise, the collective conclusions of these reviews describe mixed evidence in support of the hypothesis that tinnitus adversely impacts cognitive performance and individually included insufficient data to form conclusions regarding associations between cognitive performance and SCC in individuals with tinnitus. Several distinct cognitive functions have been implicated in this hypothesis. Previous studies have suggested that structures relating to auditory attention and efferent structures within the subcallosal region are mechanistically involved in the adverse impacts of tinnitus on cognitive performance [16]. Functional disruption to large scale neurocognitive networks has also been suggested as a mechanism [17,18] ; specifically, a hypoactive cognitive control network and hyperactive 'default mode' or 'task-negative' network.

Of the recent reviews that discuss cognitive performance via behavioural measures in tinnitus participants, there is a notable lack of overlap in the studies that met criteria for inclusion in the final reviews; e.g. Mohamad et al.[10] reviewed nine studies and Trevis et al.[17] reviewed a total of thirty-five studies (with sixteen of these concerning cognitive performance), however, only three studies were included in both reviews. Therefore, previous work has essentially investigated the association between tinnitus and cognitive performance with different datasets. Schultz et al. [19] recently reviewed the evidence for tinnitus impacting neurocognitive profiles following traumatic brain injury. They discuss cognitive performance through selective discussion of aforementioned reviews - except Andersson and Mckenna [15] – and subsequent implications within a medicolegal context. The authors highlight the current lack of and need for empirical investigation of the association between tinnitus and cognitive performance through meta-analysis. Like any statistical

For peer review only - http://bmjopen.bmj.com/site/about/guidelines.xhtml

technique, meta-analysis is only as robust as the data that is inputted. It is, therefore, essential to include as much relevant data as possible - through a comprehensive search strategy - to ensure that conclusions are based on all evidence for an effect of tinnitus on specific cognitive domains.

Tinnitus is a symptom of heterogeneous and often unknown aetiologies. It is, therefore, inherently difficult to define and specify within the context of a systematic review. Different inclusion criteria and working definitions of tinnitus are likely to significantly influence the records included within a review. For example, Trevis et al.[17] defined 'chronic tinnitus' as participants who had experienced tinnitus for at least one month. An alternative approach would be to not attempt to temporally specify a population, but rather investigate this variable through further quantitative analysis if feasible. With regards to domains of cognitive performance, the aforementioned reviews have implicated tinnitus as impacting executive attention, although the evidence is not conclusive: additional domains of cognitive performance are also potentially associated with tinnitus, including selective attention, working memory and processing speed [12,15–17]. A promising approach to foster empirically valid insights into any association between cognition and tinnitus is through evaluating and categorising tests of cognitive performance according to the theoretical constructs that they are thought to measure [20]. Webb et al. [21] describe a cross-disciplinary taxonomy for categorising cognitive performance measures (CHC-M). It utilises combined Cattell-Horn-Carroll (CHC) and Miyake theoretical elements [22,23] and includes a comprehensive taxonomical categorisation of cognitive tasks. CHC-M taxonomy will be used to organise synthesis when investigating the association between tinnitus and cognitive performance. This approach has several benefits: it is informed by the CHC 'three strata' model of cognition, which has been empirically validated through decades of research; it incorporates executive functions, a cognitive construct of particular clinical interest, facilitating translation to the clinical domain; utilisation of a pre-existing taxonomy minimises author bias (as outcome measures are not being subjectively assigned to domains of cognitive performance by authors) and enables comparison compared to 'categorisation as usual'; finally, the taxonomy provides a clear framework around which to structure synthesis of results. Associations between categorised measures of cognitive performance and tinnitus may then be subjected to meta-analysis of specific cognitive domains in order to understand which are associated with tinnitus, as well as the best estimate of any such effect. Given the suggestive nature of the evidence provided in previous reviews, we can hypothesise that there will be negative associations between tinnitus severity and cognitive performance in the broad stratum domains of executive functions, processing speed, and general short-term memory. To summarise, although several authors have reviewed the association between tinnitus and cognitive performance assessed through behavioural measures, these have been via narrative syntheses. They have discussed different sets of studies, derived from different search strategies in the absence of a unifying taxonomy. A comprehensive, quantitative investigation of the association between tinnitus and cognitive

For peer review only - http://bmjopen.bmj.com/site/about/guidelines.xhtml

performance, building on earlier efforts in this field and exploring the underlying theoretical domains of cognition involved, is therefore both necessary and timely.

The primary aim of this work is to comprehensively review the literature and synthesize relevant data to determine the associations between tinnitus and cognitive task performance in adults. If possible, a secondary examination of patient characteristics (e.g. age or gender) or commonly used patient-reported outcomes (e.g. depression or anxiety) and their influence on any association between tinnitus and cognitive performance will also be conducted.

Methods and Analysis

The methodology of this review is reported in accordance with the Preferred Reporting Items for Systematic Reviews and Meta-Analysis Protocols (PRISMA-P) checklist [24]. Specified roles of named authors are identified throughout the review protocol.

Eligibility criteria

Only published or in-press, peer-reviewed journal articles will be considered. Articles that are not written in English or have no English language translation available will be excluded as the review team does not have resource available to support translation. No date restriction will be applied.

Review inclusion criteria are specified according to Participant, Intervention (or Interest), Comparator, Outcome and Setting (PICOS) characteristics.

Participants: Studies including adults (≥18 years) with tinnitus. Studies that include both children (<18 years) and adults will be excluded, unless the adult data are reported separately.

Intervention/Interest: Tinnitus (via self-report, tinnitus severity scale, item or established tinnitus questionnaires).

Comparator: A minimum of one established measure of cognitive performance (behavioural or self-report).

Outcome measures: An association between tinnitus and cognitive performance. Where available, data for associations between tinnitus and additional potential moderator variables will be extracted, such as measures of anxiety or depression.

For peer review only - http://bmjopen.bmj.com/site/about/guidelines.xhtml

Study design: Cross-sectional, longitudinal, experimental, quasi-experimental and observational study designs will be included (only baseline data will be extracted where multiple timepoint measurements are made).

Patient and Public Involvement

There was no patient or public involvement in the development of this manuscript.

Information Sources and Search strategy

A systematic search strategy will be employed to identify completed, peer-reviewed journal articles from the following bibliographic databases: MEDLINE (via Ovid SP), EMBASE (via Ovid SP), PsycINFO (via Ovid SP), ASSIA (via ProQuest), CINAHL (via EBSCO Host), Scopus, PubMed, and Web of Science (Science and Social Science Citation Index). Initial searches were performed in February 2018. Update searches will be conducted shortly before manuscript submission.

The search terms to be used in this systematic review were identified using free text, controlled vocabularies (i.e. Medical Subject Headings - MeSH and CINAHL Headings), literature review, opinion of authors and scrutiny of test search results. The following search strategy will be used for PubMed, which will then be adapted for other databases to be searched:

("tinnitus"[MeSH] OR "tinnitus"[tiab] OR "phantom sound*"[tiab] OR "ringing"[tiab] OR "buzzing"[tiab]) AND ("cognition"[MeSH] OR "cogniti*"[tiab] OR "memory"[tiab] or "attention*"[tiab] OR "executive"[tiab])

Data management

NAC will be responsible for data management. Covidence online systematic review software (https://www.covidence.org) will be used for article screening and data management throughout the review. All articles identified through the search process will be recorded digitally and will be trackable through the data screening and extraction processes. Articles excluded at the full text screening stage will have justification for exclusion noted. Included articles will be assigned a unique study identification code, enabling it to be linked to its corresponding full text and data collection sheet.

Article selection process

NAC, DJH, and MAA will independently screen titles and abstracts of records retrieved from searches such that all records are independently screened by two reviewers. Records that meet the specified inclusion criteria will then be taken forward to full text screening, as well as records where there is too little information available to make a decision to exclude. All full texts selected will be independently screened by two reviewers who will resolve any discrepancies in which records are

For peer review only - http://bmjopen.bmj.com/site/about/guidelines.xhtml

included. Where discrepancies for inclusion are not resolved by the two reviewers, a third reviewer will adjudicate. If necessary, study authors will be contacted to request additional information that may help ascertain suitability for inclusion.

Data extraction process

Data extraction will be performed using a customised form via Covidence. Relevant guidance notes will be created and disseminated to review team members by NAC prior to commencement. The data form and guidance notes will be piloted by NAC and HH. All included records will be subject to data extraction. Data from each included record will be extracted independently by two reviewers and the results compared. Any disagreements arising will be resolved through discussion or the involvement of a third reviewer.

Data items

The data collection form will include various fields corresponding to study type, population, reported effect size, and any other relevant study findings. Study authors will be contacted and if there are relevant missing data. This will entail one email reminder, with instances of no response being reported as such. Data will be approximated from figures for instances where it can only be estimated, using software such as WebPlotDigitizer (http://arohatgi.info/WebPlotDigitizer/app/). Disagreements regarding numerical data extracted from figures will discussed by investigators and/or resolved by averaging.

Risk of bias in individual studies

NAC, HH, and DJH (two reviewers per record) will independently assess the risk of bias using the Weight of Evidence (WoE) framework, which allows appraisal of study criteria that is tailored to the review question [25,26]. The WoE framework will be customised and used to evaluate *Methodological Quality* (WoE A), *Methodological Relevance* (WoE B), and *Topic Relevance* (WoE C). A rating of low, medium, or high will be assigned for each category in accordance with WoE framework criteria. Disagreements regarding bias appraisal will be resolved through discussion or the involvement of a third reviewer.

Data synthesis

The effect of interest is the association between tinnitus and measures of cognitive performance expressed as correlation. Where possible, results not expressed as correlations (e.g. mean differences between groups) will be calculated as appropriate correlations, such as point-biserial or polyserial correlation coefficients.[27] Bias corrections will be applied to all derived correlation coefficients prior to pooling [28]. Where possible, missing effect sizes will be calculated from reported test

For peer review only - http://bmjopen.bmj.com/site/about/guidelines.xhtml

statistics such as standard deviations or t-values [27]. Narrative synthesis will be undertaken for records where appropriate effect sizes cannot be obtained. If SCC measures assess comparable constructs, a separate meta-analysis will be undertaken for the association between tinnitus and SCC. If not, these studies will be synthesised narratively. Cognitive performance will be collapsed over 'broad' factors within the level '2' stratum to enable meta-analysis of the association between tinnitus and cognitive performance these domains. If possible, 'narrow' factors within 'level 3' stratum will be meta-analysed, however, if too few records are included to afford this degree of granulation, then the narrow factors will provide a framework for narrative synthesis.

Assessment of heterogeneity

The ratio of observed variation to within-study variance will be assessed with the Q-statistic, used to test the null hypothesis of homogeneity of effect sizes. The I^2 statistic will provide a further index of heterogeneity across studies. If the apparent heterogeneity across studies exceeds 50%, potential causes of heterogeneity will be explored through subgroup analysis. The Tau-squared statistic will also be used to assess the amount of heterogeneity where a random-effects model is fitted to the data. A Baujat plot will be used as a graphical means of identifying studies that contribute excessively to any observed heterogeneity and also provide insight into potential moderating variables that contribute to heterogeneity across studies [29].

Subgroup analyses

Potential contributors to heterogeneity across studies will be explored through subgroup analysis (i.e. sensitivity analyses, moderator analysis or meta-regression). *A priori* variables of interest for subgroup analyses will include tinnitus sample characteristics (duration, laterality, intermittency), study quality and variables known to adversely impact cognitive performance, including sample age, presence of hearing impairment, presence of anxiety or depression, reported medication usage and visual acuity. Additional potential moderating variables may be identified after reviewing the literature and will be documented accordingly.

Assessment of reporting bias

The influence of reporting bias through potentially unpublished results (i.e. publication bias) will be explored via funnel plots, rank correlation test and/or Egger's regression test.

Ethics and dissemination

For peer review only - http://bmjopen.bmj.com/site/about/guidelines.xhtml

No ethical issues are foreseen in this systematic review. Reports will be guided by the Preferred Reporting Items for Systematic Reviews and Meta-Analysis guidelines [30]. Various dissemination strategies will be employed, that will likely include: journal article and PhD thesis (NAC) made available via an institutional repository, results being reported at national and international academic conferences, and public and patient engagement (e.g. articles written for relevant non-specialist audiences).

Summary

A protocol is described for a systematic review and meta-analysis to determine a best estimate of the association between tinnitus and cognitive performance in adults. The relationship between tinnitus, cognitive performance, and SSC will also be examined. To date, no review has comprehensively explored the veracity of an association between tinnitus and cognitive performance through application of quantitative analyses of all available peer-reviewed data. The outlined approach will facilitate an understanding of the potential impact of tinnitus upon cognitive performance, underpinned by relevant cognitive theory. An increased understanding of the relationship between tinnitus and cognitive performance will eventually improve tinnitus sub-typing and inform therapeutic methods[31–34] for example, it may be possible to deliver cognitive training paradigms in a targeted manner.

Acknowledgements

Thanks to Oliver Zobay for statistical advice.

References

1 Sedley W, Friston KJ, Gander PE, *et al.* An Integrative Tinnitus Model Based on Sensory Precision. *Trends Neurosci* 2016;**39**:799–812. doi:10.1016/j.tins.2016.10.004

2 Møller AR, Langguth B, DeRidder D, *et al.* *Textbook of Tinnitus*. Springer Science & Business Media 2010. https://market.android.com/details?id=book-YStcWFsxQZEC

3 Tyler RS, Baker LJ. Difficulties experienced by tinnitus sufferers. *J Speech Hear Disord* 1983;**48**:150–4. doi:10.1044/jshd.4802.150

4 Andersson G, Lyttkens L, Larsen HC. Distinguishing levels of tinnitus distress. *Clin Otolaryngol Allied Sci* 1999;**24**:404–10.

5 Bhatt JM, Bhattacharyya N, Lin HW. Relationships between tinnitus and the prevalence of anxiety and depression. *Laryngoscope* 2017;**127**:466–9. doi:10.1002/lary.26107

For peer review only - http://bmjopen.bmj.com/site/about/guidelines.xhtml

6 Srisurapanont M, Suttajit S, Eurviriyanukul K, *et al.* Discrepancy between objective and subjective cognition in adults with major depressive disorder. *Sci Rep* 2017;**7**:3901. doi:10.1038/s41598-017-04353-w

7 Petersen SE, Posner MI. The attention system of the human brain: 20 years after. *Annu Rev Neurosci* 2012;**35**:73–89. doi:10.1146/annurev-neuro-062111-150525

8 Baddeley A. Working memory. *Curr Biol* 2010;**20**:R136-40. doi:10.1016/j.cub.2009.12.014

9 Hallam RS, McKenna L, Shurlock L. Tinnitus impairs cognitive efficiency. *Int J Audiol* 2004;**43**:218–26.

10 Rossiter S, Stevens C, Walker G. Tinnitus and its effect on working memory and attention. *J Speech Lang Hear Res JSLHR* 2006;**49**:150–60. doi:10.1044/1092-4388(2006/012)

11 Heeren A, Maurage P, Perrot H, *et al.* Tinnitus specifically alters the top-down executive control sub-component of attention: evidence from the Attention Network Task. *Behav Brain Res* 2014;**269**:147–54. doi:10.1016/j.bbr.2014.04.043

12 Mohamad N, Hoare DJ, Hall DA. The consequences of tinnitus and tinnitus severity on cognition: A review of the behavioural evidence. *Hear Res* 2016;**332**:199–209. doi:10.1016/j.heares.2015.10.001

13 Robinson OJ, Vytal K, Cornwell BR, *et al.* The impact of anxiety upon cognition: perspectives from human threat of shock studies. *Front Hum Neurosci* 2013;**7**:203. doi:10.3389/fnhum.2013.00203

14 Lee RSC, Hermens DF, Porter MA, *et al.* A meta-analysis of cognitive deficits in first-episode Major Depressive Disorder. *J Affect Disord* 2012;**140**:113–24. doi:10.1016/j.jad.2011.10.023

15 Andersson G, McKenna L. The role of cognition in tinnitus. [Review] [32 refs]. *Acta Otolaryngol Suppl (Stockh)* 2006;**1**:39–43.

16 Tegg-Quinn S, Bennett RJ, Eikelboom RH, *et al.* The impact of tinnitus upon cognition in adults: A systematic review. *Int J Audiol* 2016;**55**:533–40. doi:10.1080/14992027.2016.1185168

17 Trevis KJ, McLachlan NM, Wilson SJ. A systematic review and meta-analysis of psychological functioning in chronic tinnitus. *Clin Psychol Rev* Published Online First: 2018. doi:10.1016/j.cpr.2017.12.006

18 Trevis KJ, McLachlan NM, Wilson SJ. Cognitive mechanisms in chronic tinnitus: Psychological markers of a failure to switch attention. *Front Psychol* 2016.

19 Schultz IZ, Sepehry AA, Greer SC. Impact of Tinnitus on Cognitive Function in Forensic Neuropsychology Context. *Psychol Inj Law* 2018;:1–9. doi:10.1007/s12207-018-9321-2

20 Dryden A, Allen HA, Henshaw H, *et al.* The Association Between Cognitive Performance and Speech-in-Noise Perception for Adult Listeners: A Systematic Literature Review and Meta-Analysis. *Trends Hear* 2017;**21**:2331216517744675. doi:10.1177/2331216517744675

21 Webb SL, Loh V, Lampit A, *et al.* Meta-Analysis of the Effects of Computerized Cognitive Training on Executive Functions: a Cross-Disciplinary Taxonomy for Classifying Outcome Cognitive Factors. *Neuropsychol Rev* 2018;**28**:232–50. doi:10.1007/s11065-018-9374-8

For peer review only

For peer review only - http://bmjopen.bmj.com/site/about/guidelines.xhtml

22 Miyake A, Friedman NP, Emerson MJ, *et al.* The Unity and Diversity of Executive Functions and Their Contributions to Complex "Frontal Lobe" Tasks: A Latent Variable Analysis. *Cogn Psychol* 2000;**41**:49–100. doi:10.1006/cogp.1999.0734

23 Jewsbury PA, Bowden SC, Duff K. The Cattell–Horn–Carroll Model of Cognition for Clinical Assessment. *J Psychoeduc Assess* 2016;**35**:547–67. doi:10.1177/0734282916651360

24 Shamseer L, Moher D, Clarke M, *et al.* Preferred reporting items for systematic review and meta-analysis protocols (PRISMA-P) 2015: elaboration and explanation. *BMJ* 2015;**349**:g7647. doi:10.1136/bmj.g7647

25 Gough D. Weight of Evidence: a framework for the appraisal of the quality and relevance of evidence. *Res Pap Educ* 2007;**22**:213–28. doi:10.1080/02671520701296189

26 Weed Douglas L. Weight of Evidence: A Review of Concept and Methods. *Risk Anal* 2005;**25**:1545–57. doi:10.1111/j.1539-6924.2005.00699.x

27 Lipsey MW, Wilson DB. *Practical Meta-Analysis*. SAGE Publications 2000. https://market.android.com/details?id=book-BKVpAAAAMAAJ

28 Hunter JE, Schmidt FL. *Methods of Meta-Analysis: Correcting Error and Bias in Research Findings*. SAGE 2004. https://market.android.com/details?id=book-kImkui18i94C

29 Baujat B, Mahé C, Pignon J-P, *et al.* A graphical method for exploring heterogeneity in meta-analyses: application to a meta-analysis of 65 trials. *Stat Med* 2002;**21**:2641–52. doi:10.1002/sim.1221

30 Moher D, Liberati A, Tetzlaff J, *et al.* Preferred reporting items for systematic reviews and meta-analyses: the PRISMA statement. *PLoS Med* 2009;**6**:e1000097. doi:10.1371/journal.pmed.1000097

31 Hoare DJ, Stacey PC, Hall DA. The efficacy of auditory perceptual training for tinnitus: a systematic review. *Ann Behav Med* 2010;**40**:313–24. doi:10.1007/s12160-010-9213-5

32 Baune BT, Renger L. Pharmacological and non-pharmacological interventions to improve cognitive dysfunction and functional ability in clinical depression--a systematic review. *Psychiatry Res* 2014;**219**:25–50. doi:10.1016/j.psychres.2014.05.013

33 Ferguson M, Henshaw H. How Does Auditory Training Work? Joined-Up Thinking and Listening. *Semin Hear* 2015;**36**:237–49. doi:10.1055/s-0035-1564456

34 Barrett DJK, Pilling M. Evaluating the Precision of Auditory Sensory Memory as an Index of Intrusion in Tinnitus. *Ear Hear* 2017;**38**:262–5. doi:10.1097/AUD.0000000000000367

For peer review only

For peer review only - http://bmjopen.bmj.com/site/about/guidelines.xhtml

PRISMA-P 2015 Checklist

This checklist has been adapted for use with protocol submissions to *Systematic Reviews* from Table 3 in Moher D et al: Preferred reporting items for systematic review and meta-analysis protocols (PRISMA-P) 2015 statement. *Systematic Reviews* 2015 **4**:1

Section/topic	#	Checklist item	Information reported		Line number(s)
			Yes	No	
**ADMINISTRATIVE INFORMATION**					
**Title**					
Identification	1a	Identify the report as a protocol of a systematic review	☒	☐	1
Update	1b	If the protocol is for an update of a previous systematic review, identify as such	☐	☐	N/A
**Registration**	2	If registered, provide the name of the registry (e.g., PROSPERO) and registration number in the Abstract	☒	☐	55
**Authors**					
Contact	3a	Provide name, institutional affiliation, and e-mail address of all protocol authors; provide physical mailing address of corresponding author	☒	☐	6-15
Contributions	3b	Describe contributions of protocol authors and identify the guarantor of the review	☒	☐	335
**Amendments**	4	If the protocol represents an amendment of a previously completed or published protocol, identify as such and list changes; otherwise, state plan for documenting important protocol amendments	☐	☐	N/A
**Support**					
Sources	5a	Indicate sources of financial or other support for the review	☒	☐	330-334
Sponsor	5b	Provide name for the review funder and/or sponsor	☒	☐	330-334
Role of sponsor/funder	5c	Describe roles of funder(s), sponsor(s), and/or institution(s), if any, in developing the protocol	☒	☐	330-334
**INTRODUCTION**					
**Rationale**	6	Describe the rationale for the review in the context of what is already known	☒	☐	33-50, 67-170
**Objectives**	7	Provide an explicit statement of the question(s) the review will address with reference to	☒	☐	167-170, 183-201

BioMed Central
The Open Access Publisher

For peer review only

Section/topic	#	Checklist item	Information reported		Line number(s)
			Yes	No	
		participants, interventions, comparators, and outcomes (PICO)			
**METHODS**					
**Eligibility criteria**	8	Specify the study characteristics (e.g., PICO, study design, setting, time frame) and report characteristics (e.g., years considered, language, publication status) to be used as criteria for eligibility for the review	☒	☐	183-201
**Information sources**	9	Describe all intended information sources (e.g., electronic databases, contact with study authors, trial registers, or other grey literature sources) with planned dates of coverage	☒	☐	207-215
**Search strategy**	10	Present draft of search strategy to be used for at least one electronic database, including planned limits, such that it could be repeated	☒	☐	216-218
STUDY RECORDS					
Data management	11a	Describe the mechanism(s) that will be used to manage records and data throughout the review	☒	☐	220-227
Selection process	11b	State the process that will be used for selecting studies (e.g., two independent reviewers) through each phase of the review (i.e., screening, eligibility, and inclusion in meta-analysis)	☒	☐	229-237
Data collection process	11c	Describe planned method of extracting data from reports (e.g., piloting forms, done independently, in duplicate), any processes for obtaining and confirming data from investigators	☒	☐	239-245
**Data items**	12	List and define all variables for which data will be sought (e.g., PICO items, funding sources), any pre-planned data assumptions and simplifications	☒	☐	247-254, 292-298
**Outcomes and prioritization**	13	List and define all outcomes for which data will be sought, including prioritization of main and additional outcomes, with rationale	☒	☐	266-278, 292-298
**Risk of bias in individual studies**	14	Describe anticipated methods for assessing risk of bias of individual studies, including whether this will be done at the outcome or study level, or both; state how this information will be used in data synthesis	☒	☐	257-263
DATA					
**Synthesis**	15a	Describe criteria under which study data will be quantitatively synthesized	☒	☐	265-278
	15b	If data are appropriate for quantitative synthesis, describe planned summary measures, methods of handling data, and methods of combining data from studies, including any planned exploration of consistency (e.g., I^2, Kendall's tau)	☒	☐	265-289
	15c	Describe any proposed additional analyses (e.g., sensitivity or subgroup analyses, meta-	☒	☐	291-298

BioMed Central
The Open Access Publisher

For peer review only - http://bmjopen.bmj.com/site/about/guidelines.xhtml

3

Section/topic	#	Checklist item	Information reported		Line number(s)
			Yes	No	
		regression)			
	15d	If quantitative synthesis is not appropriate, describe the type of summary planned	☒	☐	271-278
**Meta-bias(es)**	16	Specify any planned assessment of meta-bias(es) (e.g., publication bias across studies, selective reporting within studies)	☒	☐	302-303
**Confidence in cumulative evidence**	17	Describe how the strength of the body of evidence will be assessed (e.g., GRADE)	☒	☐	257-263

For peer review only

BioMed Central
The Open Access Publisher
